# Validation of Two Diagnostic Tools for the Screening of Temporomandibular Disorder

**DOI:** 10.3390/diagnostics16121815

**Published:** 2026-06-12

**Authors:** Emmanouil Sofoulis, Diana Elena Vlăduțu, Veronica Mercuț, Mihaela Ionescu, Mihaela Roxana Brătoiu, Alexandra Maria Rădoi, Ștefana Dică, Răzvan Mercuț

**Affiliations:** 1 Doctoral School, University of Medicine and Pharmacy of Craiova, 200349 Craiova, Romania; manosdent1987@hotmail.com (E.S.); stefanadica@yahoo.com (Ș.D.); 2 Department of Prosthodontics, University of Medicine and Pharmacy of Craiova, 200349 Craiova, Romania; diana.vladutu@umfcv.ro (D.E.V.); veronica.mercut@umfcv.ro (V.M.); alexandra.radoi@umfcv.ro (A.M.R.); 3 Department of Medical Informatics and Biostatistics, University of Medicine and Pharmacy of Craiova, 200349 Craiova, Romania; 4 Department of Plastic Surgery, University of Medicine and Pharmacy of Craiova, 200349 Craiova, Romania; razvan.mercut@umfcv.ro

**Keywords:** questionnaires, temporomandibular disorders, screening, Fonseca, TMD-7

## Abstract

**Background/Objectives**: Temporomandibular disorders (TMDs) constitute an umbrella term encompassing a spectrum of conditions, including pain in the temporomandibular joint (TMJ), masticatory muscle pain, and restricted mandibular movement. The objective of the present study was to translate the Fonseca and TMD-7 questionnaires into Romanian, to assess their face validity, and to determine the reliability of the Romanian-language versions for use in the diagnosis of temporomandibular disorders (TMDs) and in subsequent epidemiological and clinical studies. A secondary objective was to establish a scoring scale for the TMD-7 questionnaire. **Methods**: Both questionnaires were translated from English into Romanian and back-translated by two independent teams of translators, after which the final versions were established for face validity assessment and reliability testing. **Results**: The study demonstrated reliability according to the Cronbach’s α coefficient, with values of 0.862 for the TMD-7 questionnaire and 0.840 for the Fonseca questionnaire. The scoring scale corresponding to the Fonseca questionnaire could not be implemented for the TMD-7 questionnaire. **Conclusions**: Both Romanian-language questionnaires demonstrated high corresponding Cronbach’s α coefficients; however, for clinical use, the Fonseca questionnaire will be utilized, as the TMD-7 questionnaire does not have a scoring scale.

## 1. Introduction

The dento-maxillary system (DMS), also called the masticatory system, has a role, through its components, in the incision and grinding of food, swallowing, phonation and the expression of emotions, contributing to the fulfillment of some vital functions of the body [1]. Temporomandibular disorders (TMDs) are a heterogeneous group of conditions that affect the temporomandibular joints (TMJs), jaw muscles and related structures [2]. TMD is a generic term for various conditions such as TMJ pain, masticatory muscle pain, limitations of mandibular movements, and intra-articular functional disorders [3,4].

Three main classes of TMDs are described: joint disorders, including disorders of the intervertebral disks; conditions of the masticatory muscles and headaches associated with TMD [5]. Within each category, there are several disorders. TMDs often manifest through the presence of pain in the facial region, jaws, temples, and ear area. The pain can radiate to the surrounding areas and is frequently associated with difficulty opening the mouth and with mandibular movements [6,7]. In addition to these manifestations, DMS functions are performed with difficulty and produce discomfort [5].

According to the associated signs, symptoms, and comorbidities, TMD inevitably has a significant impact on a person’s quality of life [8,9,10] and poses a great socioeconomic burden on society [11,12].

Acute pain from TMD has the potential risk of becoming chronic and can cause increased levels of stress, pain irradiation, and associated mood and social disturbances [4,13].

Regarding the prevalence of the condition, Ahmed Yaseen Alqutaibi et al. 2025 [14], based on a review of the literature, estimated that almost a third of the global population (29.5%) is affected by TMD, with women being affected more frequently compared to men (36.7% vs. 26.7%). The prevalence among people under 18 years of age is 38.5%, compared to 34.1% among those aged 18 and over. TMDs are more common in Europe (33.8%), Asia (27.9%) and South America (27.3%), with the lowest prevalence in North America (19.4%). The most reported signs and symptoms of TMD are myalgia (37.2%), joint sounds/clicks (29.8%) and arthralgia (16.8%), with limited mouth opening/blocking being the least common (8.1%) [14].

The main accepted clinical examination of TMDs is based on the Diagnostic Criteria Protocol (DC/TMD) [15], which includes an extensive set of tests and questions. It is a test conducted by a clinician that consists of 12 questions and evaluates muscle and joint pain; opening, closing, laterotrusion and protrusion movements; headache in the last 30 days; type of occlusion; mouth opening; movements; joint noises; joint blockages; palpation pain; TMJ; and muscle pathologies [15,16]. However, the DC/TMD protocol is still too complicated and time-consuming to complete, and prior training of the examiner is required. Thus, despite the high prevalence and the existence of well-defined diagnostic criteria in the literature (DC/TMD) [15], TMD often goes undetected in primary dental care. Therefore, patients with TMD are often untreated and experience unnecessary suffering [17].

Validated screening tools can eliminate subjective decisions by providing standardized criteria that improve clinical evaluation by physicians. Thus, more accurate identification of subjects with TMD can be achieved, reducing undertreatment and improving the management of patients with TMD [18,19,20,21].

For some conditions, the risk of false positive results and overdiagnosis (and overtreatment) should be considered when choosing a screening method and sampling [18].

In medicine, questionnaires are used to collect quantitative data and information from respondents, who can be patients, their relatives, and healthcare professionals. In clinical research, data and information of interest can range from observable information about physical aspects to subjective patient evidence [22].

For TMD screening, questionnaires based on precisely formulated questions with impact on the researched condition are frequently used in clinical practice and research [23]. The responses obtained from respondents through the use of screening questionnaires are converted into a score, and a predefined scale is used to distinguish between the presence of disease “with disease” and its absence “without disease” [24]. However, some questionnaires published in the literature do not specify an assessment scale, such as TMD-7 [25] and Bruxscreen [26].

It is recommended to consider a number of factors when choosing a questionnaire for translation and validation into another language: it has been used in studies before, it presents as many correctly formulated questions as possible, and it has high levels of validity and reliability. The most important questionnaires used in TMD screening are the FAI (Fonseca Anamnestic Index) [27], SFAI (Short-Form Fonseca Anamnestic Index) [28,29], 3Q/TMD [4,30], American Academy of Orofacial Pain (AAOP) 10 Screening Questions [31], the TMD Pain Screener [17,32] and the TMD 7 [25].

These can be found in the literature in English. In order to use these questionnaires in Romanian, it is necessary to go through a scientific process, which involves translating a questionnaire from a foreign language into a local (or native) language—i.e., intended for the local population—and, subsequently, demonstrating that the translated version has an adequate level of both reliability and validity by administering it to a representative sample of the local population [24].

The objective of the present study was to translate into Romanian, to perform surface validation and to establish the reliability of the Fonseca and TMD-7 questionnaires in order to use them in subsequent epidemiological and clinical studies.

Also, based on the results obtained with the Fonseca questionnaire, an evaluation scale for the TMD-7 questionnaire will be established.

## 2. Materials and Methods

The study was carried out at the Dental Prosthetics Clinic of the Faculty of Dentistry, University of Medicine and Pharmacy of Craiova, between 1 March 2025 and 30 December 2025, and was approved by the Ethics Committee of the University of Medicine and Pharmacy of Craiova, no. 80 of 16 January 2025. For the validation of the questionnaires, several phases were performed, in accordance with the literature [24,33,34], which are detailed in the following sections.

### 2.1. Fonseca and TMD-7 Questionnaires

The Fonseca Questionnaire is a tool for TMD screening that has been validated and translated into multiple languages [35,36,37]. The questionnaire contains 10 questions, of which 3 questions refer to mandibular movements and masticatory muscles, 3 questions refer to the presence of pain in the head, neck and auricular areas, 1 question for TMJ noises, 1 question for specific manifestations of bruxism (clenching and grinding of teeth), 1 question regarding the presence of occlusal disharmonies and 1 general question about stress and anxiety. For each question, the following answers are available: No, Sometimes, and Yes. These correspond to the following scores: 0; 5 or 10 points.

The total score represents the sum of points corresponding to each question, resulting in a maximum score of 100. The interpretation of the final score is as follows: for values between 0 and 15 points, the patient does not present TMD; for values between 20 and 40 points, the patient has a mild form of TMD; for values between 45 and 65 points, the patient has a moderate form of TMD; and for values between 70 and 100 points, the patient presents a severe form of TMD.

The TMD-7 questionnaire comprises 7 questions, of which 4 questions are related to the characteristics of pain (headache, pain in the jaw area, pain in the neck area, pain in the forehead area) and 3 questions are about mandibular movements (difficulty opening the mouth, noise when opening and closing the mouth, difficulty chewing) [25]. The questionnaire includes pain characteristics and functional characteristics, providing data on the frequency of these manifestations. Subjects’ responses are scored according to the increasing frequency of their pain, according to the following scale: rarely or never, scored with 0; several times a month, scored with 1; once or twice a week, scored with 2; and almost every day, scored with 3. The final score is the sum of points per question, resulting in a sum from 0 to 21. This questionnaire does not provide a final scale for interpreting the results in terms of TMD severity.

### 2.2. Translation Phase

Both questionnaires were translated from English into Romanian and back-translated by two independent teams of translators, in accordance with international guidelines [24,38,39] for the cross-cultural adaptation of questionnaires.

Thus, two teams of translators were assigned the translation of the questionnaires from English into Romanian. The first team was composed of two students attending the Faculty of Dentistry, University of Medicine and Pharmacy of Craiova, who were in their IVth year of study, were enrolled in a Romanian teaching program, and had competences in English (Advanced Certificate in English University of Cambridge—Grade A), and the second team was composed of two students attending the Faculty of Dentistry, University of Medicine and Pharmacy of Craiova, who were in their VIth year of study, were enrolled in an English teaching program, and were native English speakers with knowledge in the field of dentistry.

For the reverse translation, from Romanian to English, two other teams were formed, respecting the same conditions.

The fifth team was composed of two professors at the Faculty of Dentistry Craiova who had competencies in English. This team reviewed all the translations and selected the prefinal questionnaires (Fonseca and TMD-7) to be validated, establishing their final form. For this purpose, the four variants were compared, and no qualitative differences between them were identified. All items were considered acceptable, based on the clarity and quality of the language used. Overall, the translated versions aligned with the original wording; therefore, the questionnaires were approved for use in the subsequent phases (validation and reliability) (Table 1 and Table 2).

### 2.3. Surface Validation Phase (Cognitive Debriefing)

The surface validation was accomplished by administering the questionnaires, in their prefinal form, in Romanian, to ten students from the Faculty of Dentistry, enrolled in the Ist year of study, who did not have in-depth knowledge of dentistry. The purpose was to determine if they completely understood the questions in the questionnaire. The students participating in this validation phase were randomly chosen on a voluntary basis. Both questionnaires were evaluated in the same meeting, first the Fonseca questionnaire, and then the TMD-7 questionnaire. After completing the questionnaires, the students answered additional validation questions and participated in a semi-structured interview aimed at ensuring that all questions were fully understood and that they did not encounter any difficulties. The prefinal form of the questionnaires was not changed after this surface validation phase; therefore, it was considered the final form of the questionnaire.

### 2.4. Reliability Phase (Pilot Study)

The reliability phase of a questionnaire is initially carried out on a pilot study group, where the questionnaires are administered to a small batch of participants, and the answers are analyzed using statistical tests.

#### Research Design

The reliability of both questionnaires was determined by examining the internal consistency, as measured by the Cronbach α coefficient, as well as by completing the questionnaires in both languages. Both questionnaires in Romanian (Fonseca and TMD-7) were completed by the subjects from the study group in the same initial session. Then, the corresponding versions translated into English were completed one month later, in another dedicated session, by the same subjects.

The questionnaires were considered acceptable in terms of consistency level if the Cronbach’s α coefficient was greater than 0.70.

The study group consisted of 25 students enrolled in the IVth year of study, with very good knowledge of the Romanian and English languages. The inclusion criteria in the study were the following: subjects 21 years of age or older (for the English teaching program) who knew both English and Romanian very well. Eligible subjects who refused to complete the questionnaires were not included in the study. Informed consent and written consent were obtained from all the participants.

### 2.5. Statistical Analysis

The statistical tests were performed using the IBM SPSS Statistics for Windows, v. 26.0 (IBM Corp., Armonk, NY, USA). Descriptive statistics were used to determine the median values and minimum and maximum values of the studied variables. The validation procedure included verification of the normality distribution (using the Shapiro–Wilk test) and the verification of internal consistency based on the Cronbach α coefficient and the intra-class correlation coefficient (ICC; with the following thresholds: values less than 0.5 represent poor reliability, values between 0.5 and 0.75 indicate moderate reliability, values between 0.75 and 0.9 indicate good reliability, while values above 0.9 reflect an excellent reliability). For each question in the questionnaires, differences in age group and gender were analyzed using the Mann–Whitney U test. The two sets of scores that come from the same participants were compared using the Wilcoxon Signed-Rank Test. Also, comparisons between the Romanian and English specific answers for each question were performed using the weighted kappa coefficient, thus assessing the agreement between the participants in both questionnaires. In all tests, a *p*-value of less than 0.05 was considered statistically significant.

## 3. Results

### 3.1. Analysis of the Pilot Study Group

The pilot study included 25 participants, 21 women and 4 men, aged between 21 and 33 years, with an average age of 23.56 ± 2.755. A Mann–Whitney U test was performed to determine if there were any age differences between women and men. The age distributions for both genders were similar, as assessed by visual inspection. The median ages for women and men were 22 years, and no statistically significant difference was identified (U = 36.500, z = −0.450, *p* = 0.695). For both questionnaires, the distribution of questions by gender reflected the fact that there were no statistically significant differences between the responses provided by women and men for the Fonseca and TMD-7 questionnaires, for both the Romanian and English versions.

### 3.2. Reliability Check

For the Romanian version, the Fonseca questionnaire had a high level of internal consistency, determined by a Cronbach α of 0.840, and the TMD-7 questionnaire had a similarly high level of internal consistency, determined by a Cronbach α coefficient of 0.862.

For the English version, the Fonseca questionnaire had a higher level of internal consistency, determined by a Cronbach α coefficient of 0.888, and the TMD-7 questionnaire had a high level of internal consistency, determined by a Cronbach α coefficient of 0.796, but slightly lower than the Fonseca questionnaire.

As indicated in Table 3, the corrected item–total correlations ranged from 0.290 (Q9) to 0.758 (Q6) for the Fonseca questionnaire in Romanian, and from 0.262 (Q10) to 0.881 (Q3) for the Fonseca questionnaire in English. Variations were recorded for the TMD-7 questionnaire as well. The corrected item–total correlations ranged from 0.422 (Q3) to 0.871 (Q2) for the TMD-7 questionnaire in Romanian, and from 0.3212 (Q3) to 0.840 (Q7) for the TMD-7 questionnaire in English. All items from both questionnaires met the recommended minimum correlation of 0.20 (Table 3).

No items should be excluded from the scales in order to improve Cronbach’s alpha. The ICCs for the individual items varied from 0.560 to 0.929 for the Fonseca questionnaire, and they were mostly excellent. Similarly, the ICCs for the individual items varied from 0.504 to 1.000 for the TMD-7 questionnaire.

These results suggest that both questionnaires demonstrate high reliability.

### 3.3. Fonseca Questionnaire Analysis

**Figure 1:**
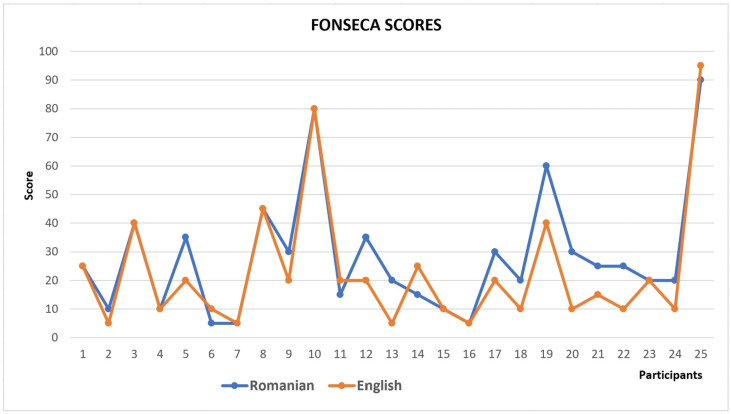
Comparison between the scores obtained with the Fonseca questionnaires in Romanian and in English.

For the Romanian version, the Fonseca score for the entire study group ranged from a minimum of 5 points to a maximum of 90 points, while the English version recorded the same minimum value, but a maximum of 95 points. Despite the differences in the overall scores obtained by each participant, highlighted in Figure 1, there were no statistically significant differences between the scores of the Fonseca questionnaires in Romanian and English, *p* = 0.473 (Table 4).

According to the overall score, the Fonseca questionnaire provides a scale with four categories to interpret the results. The thresholds are 15, 45 and 65 points. The statistical analysis of the Fonseca categories led to similar results, with no differences between the two questionnaires (*p* = 0.484; Table 4).

Many participants provided slightly different answers to identical questions when filling in the questionnaires. For example, for Question Q1 in the Romanian questionnaire, the participants filled in a total of 19 “No” answers, 5 “Sometimes” answers, and 1 “Yes” answer. For the same question, Q1, in the English questionnaires, there were 20 “No” answers, 4 “Sometimes” answers, and 1 “Yes” answer. Questions Q1 and Q3 had different answers for only one participant, while the others varied for at least two participants. For questions Q8 and Q9, two participants gave totally different answers (the overall score difference was 10), while for question Q4, only one participant gave a similar answer. The distribution of responses for the questions from the Fonseca questionnaire is shown in Table 5.

Despite the differences in the questionnaires in both languages, all ten questions were similar in terms of the overall distribution of answers, with mostly moderate to very good agreement between the Romanian and English versions. Questions with a higher percentage of participants who provided different answers had a lower weighted kappa coefficient.

**Figure 2:**
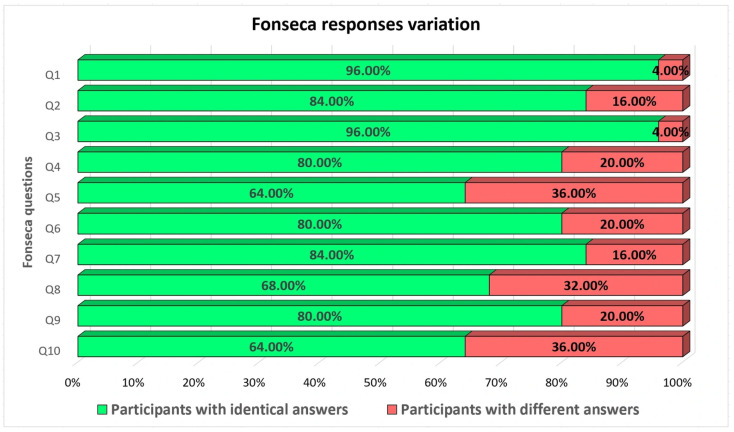
Percentage of participants with different answers to the Fonseca questionnaire.

The differences between the answers given by the participants to the two variants of the Fonseca questionnaire (Romanian and English) were defined as variations. For each question in the questionnaire, the number of participants who had variations in the answers was determined, and the overall results were displayed as percentages of participants with identical and different responses (Figure 2).

Questions Q4 and Q5 reflected rather high percentages of participants with different answers, and they referred to the reporting of head and neck pain. Question Q4 also contained the word “frequent”, indicating that the participants took into account a longer period of time when defining the answer. On the other hand, question Q5 seemed to have been interpreted at the present time, and the presence of neck pain was analyzed only at the time of the answer. There were also greater differences in question Q10, regarding the self-definition of participants as anxious people, and the answers seemed to have considered the state of the participant at the time he/she defined the answer.

Overall, the number of participants with different answers was higher only for questions Q5 and Q10, over 35%. All the other percentages were lower, indicating a general trend of similarity within the answers.

### 3.4. TMD-7 Questionnaire Analysis

**Figure 3:**
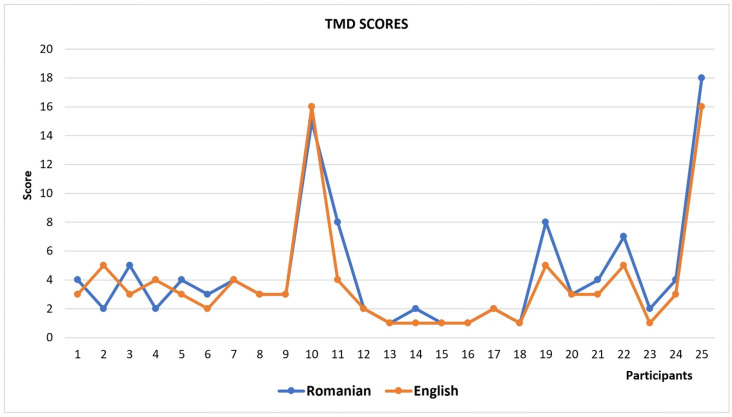
Comparison between the score obtained with the TMD-7 questionnaire in Romanian and in English.

For the Romanian version of the TMD-7 questionnaire, the score for the entire study group ranged from a minimum of 1 point to a maximum of 18 points, while the English version of the questionnaire recorded the same minimum value, and a maximum of 16 points, given that the possible answers for each question were 0 points, 1 point, 2 points and 3 points. Overall, there were fewer differences between the overall scores (Figure 3), as the median values for both questionnaires were identical, 3.00, and the Wilcoxon Signed-Rank Test revealed no statistically significant differences between the scores (*p* = 0.648; Table 6).

For TMD-7, participants also provided slightly different answers for identical questions when filling in the questionnaires in both languages. Question Q7 received the same answers for both versions of the questionnaires. For all other questions, the answers varied for at least two participants. The distribution of responses to the TMD-7 questions is presented in Table 7. Questions Q1–Q6 were similar in terms of the distribution of answers, with no statistically significant differences between the Romanian and English versions. Questions with a higher percentage of participants with different answers had a lower *p*-value but were still above the threshold of statistical significance.

**Figure 4:**
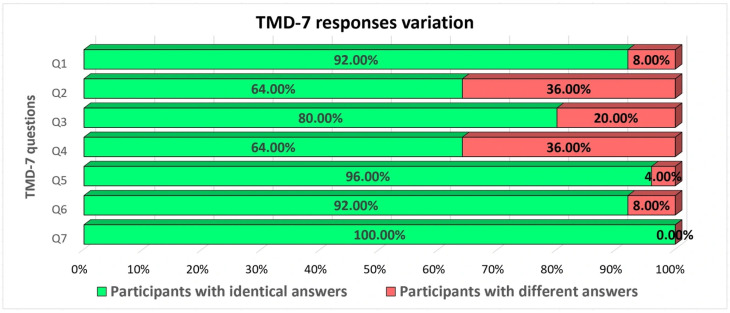
Percentage of participants with different responses to the TMD-7 questionnaire.

Similar to the Fonseca questionnaire, for each question in the TMD-7 questionnaire, the number of participants who had variations in the answers was determined, and the overall results were displayed as percentages of participants with identical and different responses (Figure 4).

Questions Q2, Q3 and Q4 had higher values of participants with different answers (at least 20%) and referred to reporting pain in the head, neck, shoulders, jaws or ears. Thus, the answers seem to have been interpreted at the moment of filling in the questionnaire, and the presence of pain was analyzed only at the time of the answer. All the other percentages were lower, below 10%, indicating thus a general trend of similarity within the answers.

### 3.5. Definition of a Rating Scale for the TMD-7 Questionnaire

For both the Fonseca and TMD-7 questionnaires, the overall score is computed by adding the individual points of each question. For Fonseca scores, the potential values may be categorized into four categories of severity of TMD (No TMD, Mild TMD, Moderate TMD, and Severe TMD). Given the fact that the participants in the pilot study filled in both questionnaires, the Fonseca categories and the potential equivalent TMD-7 categories can be mathematically mapped according to Table 8.

**Figure 5:**
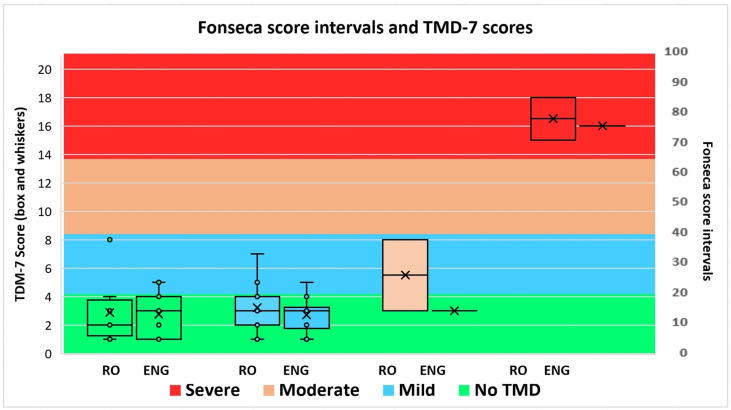
Graphical representation of TMD-7 scores (vertical left axis, box and whiskers representation) for participants in the study group, compared to Fonseca intervals (vertical right axis, background intervals). The green range represents the “No TMD” category and corresponds to the range 0–15. The blue range represents the “Mild TMD” category and corresponds to the range 20–40. The orange range represents the “Moderate TMD” category and corresponds to the range 45–65. The red range represents the “Severe TMD” category and corresponds to the range 70–100. The colors of the TMD-7 representations are defined according to the category mapping between the two questionnaires.

According to Figure 5, for the Romanian version, participants in the Fonseca “No TMD” category recorded TMD-7 scores in the range 1–4, with one exception—a value of 8; for the English version, participants in the Fonseca “No TMD” category recorded TMD-7 scores in the range 1–5. The “Mild TMD” category was represented as follows: for the Romanian version, the participants recorded TMD-7 scores in the range 1–5, with only one exception value—7—and for the English version, the participants recorded TMD-7 scores in the range 1–5. The “Moderate TMD” category was represented as follows: for the Romanian version, the two participants recorded TMD-7 scores of 3 and 8, and for the English version, the only participant in this category recorded a TMD-7 score of 3. Two participants fell into the “Severe TMD” category: for the Romanian version, the TMD-7 scores 15 and 18 were recorded, and for the English version, both participants in this category recorded a TMD-7 score of 16.

## 4. Discussion

TMD is the most common cause of chronic orofacial pain [17]. Screening can ensure equality in healthcare, as unbiased tools could help minimize gender and age-related inequalities [18]; at the same time, early detection of TMD can help prevent the chronicization of pain and reduce its impact on the individual and society [20,21]. Therefore, effective and reliable screening is a critical step in identifying TMD in the general population, as the literature shows a wide variation in the prevalence and symptoms of TMD in different populations.

The approach to TMD has changed over time based on scientific evidence that has emerged from a consequence of occlusal disorders to a complex disorder within a biopsychosocial disease model, confirming that for almost all cases, a multidisciplinary approach is needed.

After describing “Costen’s syndrome”, dental professionals were interested in its symptoms but questioned the etiology and proposed treatment [3]. The treatment proposed for TMJ pain consisted of using devices that “increase the occlusal vertical dimension” described by Costen himself [3,40], and from this period, dental occlusion and especially occlusal interference were considered an etiological factor of TMD [41].

The scientific study of the correlation between dental occlusion and TMD began after the 1950s with electromyographic studies of masticatory muscles [42]. It was also during this period that the first disorders of the masticatory muscles caused by occlusal disharmonies were described [43].

Between the 1960s and 1970s, dental occlusion and emotional stress were considered major etiological factors of functional disorders of DMS, and the interest of professionals in these disorders increased. It was also during this period that information emerged about painful disorders arising from inside the TMJ [44].

After the 1980s, the complexity of the manifestations including TMD [45] and the impact of orofacial pain on the functionality of DMS and the human body in general [46] were fully understood.

After the introduction of the concept of evidence-based medicine between 1990 and 2000, there was a need for more in-depth research in establishing diagnosis, treatment and organizing training programs to better prepare clinicians for the management of patients with TMD. The diagnostic research criteria for TMD (DRC/TMD) were proposed in 1992 [47] out of the need to establish a diagnostic system that would reliably distinguish, for epidemiological and clinical research purposes, cases of TMD and define and differentially establish the common subtypes of TMD specific to chronic pain.

The DRC/TMD proposed in 1992 by Dworkin and LeResche was the first step toward a complex diagnostic system and consists of a questionnaire with 31 questions on general health, facial pain, mandibular dynamics, facial trauma, bruxism-specific manifestations, social and psychological status, demographic data and education level [47].

The basic principles underlying this diagnostic approach included: a biopsychosocial model to assess and classify the disease; epidemiological data for discerning the distribution of signs and symptoms by gender and age and for identifying population norms from which the disease could be better defined; a two-axis diagnostic system composed of the physical diagnosis (Axis I) and the psychosocial profile (Axis II); the strict definition of operational terms, including the establishment of precise specifications for clinical examination and protocols for the necessary reliability and validation studies; and recognition that on the basis of evidence, protocols will require inevitable subsequent revisions [48].

Later, in Axis I, in addition to clinical evaluation, radiological evaluation was also introduced, and it is designed to differentiate myofascial pain, disk displacement, and arthralgia-arthritis and arthrosis. Axis I of the DRC/TMD briefly described the image analysis criteria for highlighting TMJ disk displacement by arthrography and magnetic resonance imaging (MRI) and osteoarthritis based on computed tomography (CT) [47]. Although panoramic radiography was not included as an imaging option in the initial system of DRC/TMD, it was recommended as a screening tool for TMJ pathology [49]. Once the use of CT and MRI was higher, it was necessary to develop comprehensive criteria for image analysis using these techniques for DRC/TMD [50]. The RDC/TMD validation has shown that for radiological examination, MRI is not an ideal imaging technique for detecting bone changes, so CT remains the image of choice for bone tissue evaluation. Compared to soft-tissue assessment, MRI has excellent reliability for assessing disk position.

Axis II assesses psychological status and pain-related disability. Following these parameters, the critical review of the diagnostic systems in existence at the time revealed considerable shortcomings and substantiated data in support of the development of CPR/TDG research standards [48]. The DRC/TMD did not contain research in the developing areas of genetics, neuroscience, and diagnostic tools to quantify the relationships between the subjective ratio of pain and physiological findings [51]. Based on the results obtained from the review of the existing diagnostic systems in the literature, the major basic objectives that must underpin the DRC/TMD approach have been formulated: the development of a diagnostic system based on the presence of self-reported pain [52], a minimum clinical examination to obtain case definitions and reliable diagnoses, and obtaining sufficient data through which the limitations of DRC/TMD could be identified [53]. In order to achieve these objectives, scientific societies with recognized research activity were involved, namely the “American Dental Association”, “National Institute of Dental and Craniofacial Research”, and “International Association for Dental Research”.

The next stage after the elaboration of the DRC/TMD [47] and its use in various scientific research was the validation of this system [54]. Some deficiencies of the DRC/TMD were noted.

Within Axis I, there is no differentiation between TMJ pain and other painful facial conditions. The immediate recommendation was to apply DRC/TMD after other sources of pain, including odontogenic pain, were excluded; the nomenclature of the RDC/TMD should be changed to the Diagnostic Criteria for TMD (DC/TMD), thus encouraging practitioners to use this diagnostic system. Also, regarding the nomenclature, it was observed that practitioners use different terms: for group I of muscle disorders, in addition to myofascial pain, the term myalgia was proposed when an area triggering the pain was not detected. However, the term myalgia is not used by practitioners. For group II, in the case of disk displacements, it was recommended that those displacements that alter the mandibular dynamics should be called “internal disturbances”, and the term “disk displacement” should designate benign states when these situations cannot be clinically detected or have no clinical consequences. For group III, the term arthralgia is recommended rather than arthritis or arthrosis because clinical signs of inflammation, other than joint pain on palpation and loss of function, such as local heat, erythema and swelling, are rarely observed in the TMJ. It has also been suggested to use the term degenerative joint disease instead of osteoarthritis and osteoarthrosis.

The RDC/TMD published in 1992 showed an acceptable threshold for diagnostic validity as a sensitivity level of at least 0.70 and a specificity greater than 0.95 [54].

Within Axis II, the assessment of psychosocial dysfunction based on depression is an extremely important aspect. Along with the assessment of suffering, the potential for self-harm is also assessed. The low specificity of depression screening indicates that it needs to be improved.

In parallel with these efforts to develop a diagnostic system, other specialists have also published questionnaires and examination protocols for TMD [55,56,57].

As a result of this analysis, DC/TMD [15] was proposed, which evaluates common TMD and has clinical and research utility; extended DC/TMD [58] for rare TMD, DC/TMD and DC/TMD have been published in extended form by the American Academy of Orofacial Pain [59], and a summary of DC/TMD has been produced [60] to better disseminate diagnostic standards in the general clinical field. In order to make a more correct diagnosis and to reduce the risk of diagnostic errors, it is necessary to correlate the answers obtained on Axis I and Axis II. However, in order to make an immediate diagnosis, it is necessary to select questions that do not confuse the patient in terms of the answers to the questions [61].

The diagnostic stage is very important for both doctors and people living with TMD [62]. It has been found that establishing the diagnosis with the delay or lack thereof directly affects the severity of symptoms and the quality of life of a person [63]. For those affected, the diagnosis provides an explanation for the suffering, the assumption of the condition and a justification to themselves and to family, friends and co-workers [64].

In this regard, specialists in the field have proposed questionnaires through which questions can capture a series of symptoms specific to TMD based on self-report [32]. If the results are positive, then a more comprehensive evaluation of the respective patients is required.

Quizzes are also known as scales and tools [23]. The purpose of using a questionnaire is to obtain fast, simple and reliable information that will be of assistance to doctors and the patient. Several features of DMS function disorders and pain can be identified by self-reporting by the patient. Lately, there has been an increase in the use of questionnaires and types of questionnaires. Axel Kutschke et al., based on a 2025 review, showed that for TMD, they identified 21 different screening tools [17].

The Fonseca Anamnestic Index (FAI) is a 10-question questionnaire, designed in 2006 [65] and 1994 [27], based on the Helkimo Anamnestic Index [17,66]. The FAI is recommended for TMD screening in public health facilities because it is cost-effective and quick and easy to apply [67]. For this tool, Bernie et al. reported high sensitivity and specificity for myogenic TMDs in women [68], and Kaynak et al. reported high sensitivity and specificity in participants from a university community in Turkey [69]. Other studies using FAI have reported that FAI is highly sensitive but has low specificity, resulting in the questionnaire being good for diagnosing TMDs but failing to identify people who do not have TMD [17,70,71,72]. However, the Fonseca questionnaire has been used in several studies to determine the prevalence of TMD [16,36,37,71,73,74].

The TMD-7 questionnaire was developed over a period that started in November 2019 and ended in May 2020 [25]. It includes pain (questions 1–4) and functional (questions 5–7) characteristics. The goal of TMD-7 was to provide a short measuring instrument for the patient to fill in that provides enough information to take a patient into treatment. Koufos EB reported for the TMD-7 questionnaire a Cronbach α coefficient of 0.77 [25].

The evaluation of the questionnaires is carried out through internal consistency, which is a measure of the intercorrelation of the questionnaire items and, therefore, of the consistency in measuring the targeted problem. The commonly used method for measuring internal consistency is by calculating the Cronbach α coefficient [22].

For a given questionnaire, the Cronbach value usually ranges from 0 to 1 and can sometimes be negative if some items are negatively correlated with other items in the questionnaire. The zero value of Cronbach’s α coefficient indicates a lack of internal consistency (i.e., the questions in the questionnaire are not correlated with each other). Increasing the positive value of the Cronbach α coefficient above zero shows that the questions are more strongly interconnected with each other. The value of the Cronbach coefficient for a questionnaire equal to one indicates perfect internal consistency (i.e., all questionnaire questions are perfectly correlated with each other). According to experts’ suggestions, the value of Cronbach’s α coefficient is expected to be at least 0.70 to indicate adequate internal consistency of a given questionnaire. A low value (below 0.7) of the Cronbach’s α coefficient for a given questionnaire represents poor internal consistency and therefore poor interconnection between elements. It has been found that the value of the Cronbach α coefficient is a function of the length of the questionnaire (i.e., the number of questions in the questionnaire) and increases as the number of questions increases.

The present study aimed to validate the Romanian versions of two questionnaires recommended for TMD screening. Going through the necessary steps to establish reliability, it was found that both translated questionnaires have a Cronbach’s α coefficient above 0.7, so they are suitable for use in clinical trials. The difference between the questionnaires results from the number of questions and the fact that the TMD-7 questionnaire does not have a scoring scale. Relating the results obtained with TMD-7 to the Fonseca results, it was found that TMD-7 underdiagnoses cases with TMD, especially mild and medium forms.

Taking into account the rating scale of the Fonseca questionnaire, considered the gold standard, it is found that the TMD-7 questionnaire underdiagnoses mild and moderate forms, since it manages to diagnose only severe forms of TMD. Therefore, for the following research, we recommend the use of the Fonseca questionnaire, although the TMD-7 questionnaire seems easier to translate (having many technical terms) and to use (having fewer questions).

According to the results obtained, the Fonseca questionnaire in Romanian will be used further, and its reliability will be determined in field research by applying the same statistical tests on a larger sample.

This study had the following limitation: it was performed with a small, gender-imbalanced sample of dental students, all with prior knowledge of TMD, given their current training as future dentists.

## 5. Conclusions

The objectives of the study were to translate the Fonseca Questionnaire and the TMD-7 Questionnaire into Romanian to be used in TMD screening, as well as to define an evaluation scale for the TMD-7 questionnaire.

Regarding the reliability of the two questionnaires, for the Romanian versions, both questionnaires had a high level of internal consistency, with the TMD-7 questionnaire scoring slightly higher.

Based on the absence of a scale for evaluating the final score and the mismatch between the results obtained with the two questionnaires, we recommend the Fonseca questionnaire for subsequent studies.

## Figures and Tables

**Table 1:** Fonseca questionnaire in English and Romanian.

Q *	English Version	Romanian Version
Q1	Do you have difficulty opening your mouth wide?	Deschizi gura cu dificultate?
Q2	Do you have difficulty moving your jaw to the sides?	Iți este greu să miști mandibula dintr-o parte în alta?
Q3	Do you feel fatigue or muscle pain when you chew?	Ai dureri ale mușchilor masticatori în timpul masticației?
Q4	Do you have frequent headaches?	Ai des dureri de cap?
Q5	Do you have neck pain or stiff neck?	Ai dureri în zona gâtului sau o stare de contractură a gâtului?
Q6	Do you have earaches or pain in your temporomandibular joint?	Ai dureri în zona auriculară?
Q7	Have you ever noticed any noise in your temporomandibular joint while chewing or opening your mouth?	Ai auzit zgomote în articulație atunci când mesteci sau deschizi gura?
Q8	Do you have any habits such as clenching or grinding your teeth?	Ai obiceiul de a încleșta sau scrâșni dinții?
Q9	Do you feel that your teeth do not come together well?	Simți ca dinții nu se potrivesc atunci cand închizi gura?
Q10	Do you consider yourself a tense (nervous) person?	Te consideri o persoană anxioasă sau stresată?

* Q—question.

**Table 2:** TMD-7 Questionnaire in English and Romanian languages.

Q *	English Version	Romanian Version
	How Often Are You Bothered by Any of the Following Problems…	Cât de Des te Simți Deranjat de Oricare dintre Următoarele Tulburări?
Q1	Headache	Durere de cap
Q2	Pain in your jaw or ears	Durere în zona maxilarelor sau în zona urechilor
Q3	Pain in your neck or shoulders	Durere la nivelul gâtului sau umerilor
Q4	Pain in your forehead or temples	Durere la nivelul frunții sau tâmplelor
Q5	Difficulty opening your mouth all the way	Dificultăți la deschiderea largă a gurii
Q6	Noise when opening or closing your mouth	Zgomote în deschiderea/închiderea gurii
Q7	Difficulty when eating or chewing your food	Dificultăți în timpul masticației

* Q—question.

**Table 3:** Internal consistency and reliability for the Fonseca questionnaire.

Question	Corrected Item–Total Correlation		Cronbach’s Alpha If Item Deleted		ICC
	Romanian	English	Romanian	English	
**FONSECA questionnaire**					
Q1	0.735	0.802	0.811	0.867	0.929
Q2	0.653	0.716	0.818	0.872	0.695
Q3	0.714	0.881	0.812	0.858	0.944
Q4	0.650	0.675	0.813	0.873	0.672
Q5	0.440	0.449	0.835	0.889	0.654
Q6	0.758	0.819	0.802	0.864	0.642
Q7	0.500	0.571	0.829	0.881	0.855
Q8	0.292	0.446	0.852	0.891	0.701
Q9	0.290	0.726	0.853	0.869	0.639
Q10	0.653	0.262	0.822	0.898	0.560
**TMD-7 questionnaire**					
Q1	0.571	0.418	0.850	0.788	0.927
Q2	0.871	0.784	0.805	0.726	0.727
Q3	0.422	0.321	0.879	0.811	0.767
Q4	0.523	0.483	0.856	0.781	0.504
Q5	0.681	0.737	0.839	0.741	0.953
Q6	0.614	0.414	0.846	0.822	0.659
Q7	0.855	0.840	0.813	0.716	1.000

**Table 4:** Distribution of the study group according to the results obtained with the Fonseca questionnaire for both versions, in Romanian and in English.

Parameter	Category	Questionnaire (25 Participants)		*p* *
		Romanian	English	
Fonseca score (median value)	-	25.00	20.00	0.473
Fonseca Categories	No	8 (32.00%)	12 (48.00%)	0.484
	Mild	13 (52.00%)	10 (40.00%)	
	Moderate	2 (8.00%)	1 (4.00%)	
	Severe	2 (8.00%)	2 (8.00%)	

* Wilcoxon Signed-Rank Test.

**Table 5:** Distribution of the scores obtained with the Fonseca questionnaire for both versions, in Romanian and English.

Question	Romanian			English			K *
	No	Sometimes	Yes	No	Sometimes	Yes	
Q1	19	5	1	20	4	1	0.904
Q2	20	4	1	20	4	1	0.596
Q3	18	6	1	18	5	2	0.922
Q4	13	8	4	13	10	2	0.663
Q5	9	11	5	11	12	2	0.507
Q6	18	4	3	21	2	2	0.548
Q7	17	4	4	14	8	3	0.777
Q8	12	8	5	16	6	3	0.485
Q9	18	2	5	18	3	4	0.599
Q10	3	16	6	6	17	2	0.383

* Weighted kappa.

**Table 6:** Distribution of the study group according to the results obtained with the TMD-7 questionnaire for both versions, in Romanian and in English.

Parameter	Category	Questionnaire (25 Participants)		*p* *
		Romanian	English	
TMD score	-	3.00	3.00	0.648

* Wilcoxon Signed-Rank Test.

**Table 7:** Distribution of the scores obtained with the TMD-7 questionnaire for both versions, in Romanian and English.

Question	Romanian				English				K *
	0	1	2	3	0	1	2	3	
Q1	9	10	6	0	8	12	5	0	0.899
Q2	18	5	0	2	20	3	1	1	0.427
Q3	5	12	4	4	6	14	2	3	0.713
Q4	10	12	3	0	16	8	1	0	0.410
Q5	22	2	0	1	21	3	0	1	0.898
Q6	17	5	1	2	18	4	0	2	0.678
Q7	21	2	1	1	21	2	1	1	1.000

* Weighted kappa.

**Table 8:** Fonseca and TMD-7 category mapping.

Fonseca	Category	TMD-7
0–15	No TMD	0–4
20–40	Mild TMD	5–9
45–65	Moderate TMD	10–13
70–100	Severe TMD	14–21

## Data Availability

The data presented in this study are available on request from the corresponding authors due to privacy, legal, and ethical restrictions.

## Abbreviations

The following abbreviations are used in this manuscript:

DMS	Dento-maxillary system
TMD	Temporomandibular disorders
TMJ	Temporomandibular joints
DC/TMD	Diagnostic Criteria Protocol
WHO	World Health Organization
DRC/TMD	Diagnostic Research Criteria
MRI	Magnetic resonance imaging
CT	Computed tomography
FAI	Fonseca Anamnestic Index
